# Effect of Heat Input on the Hydrogen Embrittlement Sensitivity of CGHAZ of X60 Pipeline Steel

**DOI:** 10.3390/ma19050961

**Published:** 2026-03-02

**Authors:** Longwei Zhang, Zhongwen Wu, Wenhao Zhou, Qingxue Zhang, Ba Li, Zhihui Zhang, Bing Wang, Qingyou Liu, Shujun Jia, Shubiao Yin

**Affiliations:** 1 Faculty of Metallurgy and Energy Engineering, Kunming University of Science and Technology, Kunming 650093, China; 2 Institute for Structural Steels, Central Iron and Steel Research Institute Co., Ltd., Beijing 100081, China; 3 Hunan Valin Xiangtan Iron and Steel Co., Ltd., Xiangtan 411101, China

**Keywords:** X60 pipeline steel, hydrogen embrittlement susceptibility, welding heat input, coarse grain heat-affected zone, thermal simulation

## Abstract

In the coarse grain heat-affected zone (CGHAZ) of welded pipe steel joints, hydrogen damage is a key factor limiting the high-pressure hydrogen transportation performance of the pipeline. This study employed multi-dimensional characterization methods (including microstructure, mechanical properties, and hydrogen distribution) to investigate the influence of welding heat input on the hydrogen embrittlement (HE) sensitivity of X60 pipeline steel in the CGHAZ. The results showed that as the heat input increased, the grains in the CGHAZ became coarser, and the microstructure changed from bainitic ferrite (BF) to granular bainite (GB) and polygonal ferrite (PF). Among them, the BF + GB composite structure had the best resistance to HE (HE sensitivity was 29.8%). At low heat input, the reversible hydrogen distribution occurred at the interfaces between the grain boundaries and the BF blocks, while at high heat input, it would accumulate around the martensite/austenite (M/A) constituents. For the 16 kJ/cm heat input experimental steel, the increase in Σ3 grain boundary density accelerated hydrogen diffusion and reduced its enrichment, thereby resulting in the lowest HE sensitivity.

## 1. Introduction

Hydrogen gas, as a clean energy source, has received more attention due to its ability to generate a large amount of energy upon combustion, producing only water as a by-product and having the least impact on the environment [1]. Pipeline transportation of hydrogen is the most economical and convenient method [2]. With the widespread application and research of hydrogen energy, low-strength pipeline steel can no longer meet the increasing demand for high-pressure hydrogen transportation. High-grade pipeline steel with high hydrogen embrittlement (HE) sensitivity is prone to hydrogen damage during service. The coarse-grained heat-affected zone (CGHAZ) of the welding joint is the most vulnerable part of pipeline steel. The safety and integrity of the welding joint CGHAZ in pipeline steel operating in a high-hydrogen environment face greater challenges [3,4].

Han et al. [5] utilized welding thermal simulation technology to investigate the sulfide stress corrosion cracking (SSCC) behavior of CGHAZ under different heat inputs. By observing the microstructure and fracture morphology of the experimental steel, it was concluded that the heat input has a significant impact on the microstructure and properties of the material. When t_8/5_ was 30 s, the microstructure was a lamellar bainite (LB) and granular bainite (GB) structure, which had low HE sensitivity and high SSCC resistance. Gou et al. [6] used impact tests and electrochemical hydrogen permeation experiments to study the impact fracture behavior of X80 pipeline steel weld seam CGHAZ before and after hydrogen charging. The study showed that as the heat input increased, the microstructure of CGHAZ changed from LB to GB. Compared with LB and GB, needle-like ferrite had higher anti-HE performance. The H atom lowers the threshold for brittle fracture to occur. Yan et al. [7] used thermal simulation methods to study the relationship between microstructure and HE sensitivity of X65 steel at different t_8/5_ times, and found that, compared with BF, martensite, and needle-like ferrite, needle-like ferrite had the most excellent anti-HE performance. Zhang et al. [8] conducted HE sensitivity research on the heat-affected zone (HAZ) of X80 steel using high-pressure hydrogen slow strain-rate tensile experiments, hydrogen permeation experiments, and microstructure analysis. The study found that martensite/austenite (M/A) components in BF would exacerbate the HE sensitivity of the material. M/A, as a brittle hard phase, is easily the starting point for crack initiation, leading to HE. Previous studies have all believed that, in the CGHAZ of experimental steel under high hydrogen environment with higher welding heat input, cracks would initiate and propagate along the M/A interface with the matrix. Regarding the hydrogen damage mechanism, there are currently hydrogen-enhanced decohesion (HEDE) [9], hydrogen-enhanced local plasticity (HELP) [10], hydrogen internal pressure (HIP) [11], adsorption-induced dislocation emission (AIDE) [12,13], and the hydrogen-enhanced strain-induced vacancy (HESIV) [14], among others. However, none of the current theories can fully explain all hydrogen damage phenomena [15,16]. Previous studies have mostly focused on the correspondence between microstructure, M/A components, inclusions, and the HE sensitivity of the pipeline steel CGHAZ. This study found that the density of Σ3 grain boundaries has a key influence on the HE sensitivity of the pipeline steel CGHAZ.

This paper simulates the CGHAZ microstructure through welding thermal simulation technology. The microstructure evolution of the experimental steel was investigated by using experimental and characterization techniques such as slow strain-rate testing (SSRT), low-temperature impact, scanning electron microscopy (SEM), electron back-scattering diffraction (EBSD), and hydrogen micro-print techniques (HMT), and the mechanical properties and HE sensitivity change mechanism of the experimental steel were analyzed. This research aims to provide ideas and scientific basis for enhancing the hydrogen corrosion resistance of the heat-affected zone in X60 pipeline steel during welding.

## 2. Materials and Methods

### 2.1. Materials

The test material was the X60 pipeline steel base metal (BM) prepared in the laboratory. The experimental steel was designed and prepared by Institute for Structural Steels, Central Iron and Steel Research Institute Co., Ltd., Beijing, China. Its chemical composition table is shown in Table 1. The basic mechanical properties of BM were tested in accordance with the standard GB/T228.1-2021 [17]. In this study, to ensure the accuracy of the experiment, three parallel specimens were prepared for the tensile test of the experimental BM. Table 2 shows the basic mechanical properties of BM.

### 2.2. Welding Thermal Simulation

**Figure 1:**
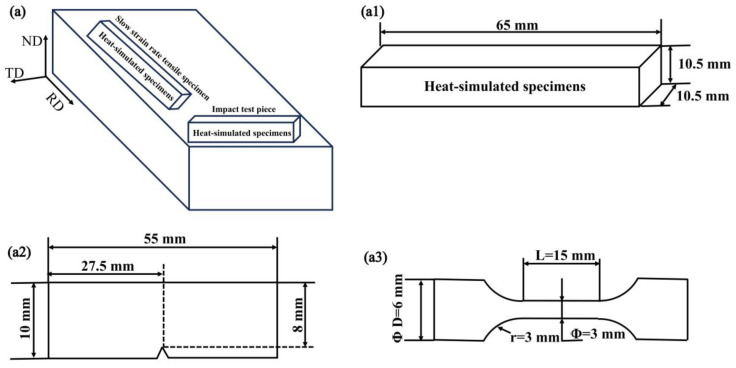
Sampling schematic diagram. (**a**) Sampling direction, (**a1**) size of the thermal simulation sample, (**a2**) size of the low-temperature impact toughness sample, (**a3**) size of the SSRT sample.

**Figure 2:**
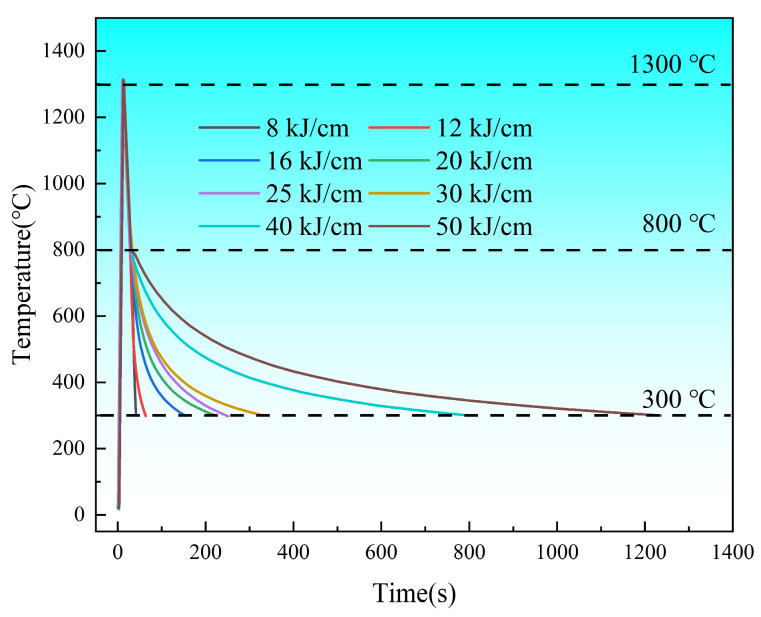
Thermal simulation image.

Before the experiment, the Gleeble 3800 (Poestenkill, NY, USA) thermal simulation testing machine was calibrated. The dimensions of the steel used in the thermal simulation experiment were 65 × 10.5 × 10.5 mm. In this study, a R-type platinum–rhodium alloy thermocouple was used to control the temperature of the experimental steel. The thermal simulation samples were heated at a rate of 130 °C/s to 1300 °C, held for 1 s, and then cooled to 800 °C. Subsequently, different heat inputs were applied respectively to cool down to 300 °C and finally cooled naturally to room temperature. Based on previous research and industrial application conditions [5,6,7], relatively appropriate heat inputs of 8, 12, 16, 20, 25, 30, 40, and 50 kJ/cm were selected. The sampling schematic diagram is shown in Figure 1(a1), and the thermal simulation curve is shown in Figure 2.

### 2.3. Microstructure

Metallographic experimental steel samples of 10 × 10 × 5 mm were taken and ground respectively with 320#, 600#, and 1000# sandpapers and then polished with 0.25 μm diamond metallographic polishing agent. The polished experimental steel was immersed in a 4% nitric acid alcohol solution for etching treatment for 8 s. Finally, its microstructure was observed using an optical microscope (OM, Olympus GX51 Olympus Corporation, Tokyo, Japan) and a scanning electron microscope (SEM, FEI Quanta 650FEG Thermo Fisher Scientific, Waltham, MA, USA). The experimental steel was corroded with a 6% perchloric acid alcohol solution and electrolytically polished for 15 s under a 20 V voltage. Subsequently, the crystallographic characteristics of the experimental steel were analyzed using a SEM equipped with an electron backscatter diffractometer (EBSD, FEI Quanta 650FEG Thermo Fisher Scientific, Waltham, MA, USA) with a scanning step size of 0.8 μm.

### 2.4. Hardness and Low-Temperature Impact

According to the requirements of ISO6507-1 standard in NACE MR0175 [18] regarding hardness measurement, the micro-Vickers hardness of the experimental steel was measured. The specific operation steps were as follows: Using the MH-500D (Hengyi, Shanghai, China) semi-automatic micro-Vickers hardness tester, apply a load of 500 g to the experimental steel with different heating amounts for 15 s. In the thermal simulation experiment, seven points in the center of the steel will be selected for hardness testing. After eliminating the highest and lowest values, the average of the remaining five values will be taken. For the low-temperature impact test, 55 × 10 × 10 mm impact specimens in accordance with the GB/T 229-2020 standard [19] were prepared. The sampling diagram is shown in Figure 1(a2). The low-temperature impact tests were conducted in a −20 °C environment. For the experimental steel with different heat inputs, three impact specimens respectively were prepared to complete the low-temperature impact test.

### 2.5. Slow Strain Rate Tensile Test

The slow strain-rate tensile specimens of experimental steel were prepared by using the standard GB/T 39039-2020 [20] evaluation method for hydrogen-induced delayed fracture of high-strength steel. The sampling diagram is shown in Figure 1(a3). The SSRT was conducted using the WDLM-3-30KN-type (Li Chuang, Xi’an, China) slow tensile testing machine. Different heat input experimental steels were subjected to two parallel experiments at room temperature in the nitrogen atmosphere, with a slow strain rate of 1 × 10^−6^ s^−1^ and 6.3 MPa of pressure. Under the same stretching rate and pressure conditions, hydrogen was first introduced for 1 h, and then three parallel experiments were conducted in the hydrogen environment. The cross-sectional diameter was measured using a microscope, and the average value was taken to calculate the fracture area. The HE sensitivity was expressed through the reduction rate and loss rate of the experimental steel cross-section. The calculation formula is as follows:

(1) $$Z=\frac{A_{0}-A_{1}}{A_{0}}$$

(2) $$I_{\mathrm{HE}}=\frac{Z_{N}-Z_{H}}{Z_{N}}$$

In the formula, A_0_ and A_1_ respectively represent the cross-sectional areas of the experimental steel before and after stretching, Z_N_ and Z_H_ represent the reduction in area in hydrogen and nitrogen environments respectively, and I_HE_ is the reduction in area loss rate, that is, the HE sensitivity.

### 2.6. Hydrogen Micro-Print Technique (HMT)

The distribution of hydrogen on the surface of experimental steel can be observed by using the hydrogen micro-print technique. This study was conducted to understand the enrichment of reversible hydrogen on the surface of the experimental steel. Three solutions were prepared in advance. First, solution I was prepared by adding 0.5 g of AgBr to 40 mL of a 5 wt% NaNO_2_ solution. Solution II was a 40% CH_2_O solution. Solution III was prepared by adding 16.8 g of 5H_2_O·Na_2_S_2_O_3_ and 10.7 g of NaNO_2_ to 100 mL of distilled water. Electrochemical hydrogen filling was carried out in 3.5 wt% NaCl solution using IT-6411S DC (ITECH, Nanjing, China) power supply at a current density of 5 mA/cm^2^ for 8 h. The distribution of silver particles on the surface of the experimental steel was observed under a scanning electron microscope. The reaction principle is shown in Equation (3):

Ag^+^ + H = Ag ↓ + H^+^ (3)

## 3. Results

### 3.1. Microstructure of X60 Pipeline Steel

**Figure 3:**
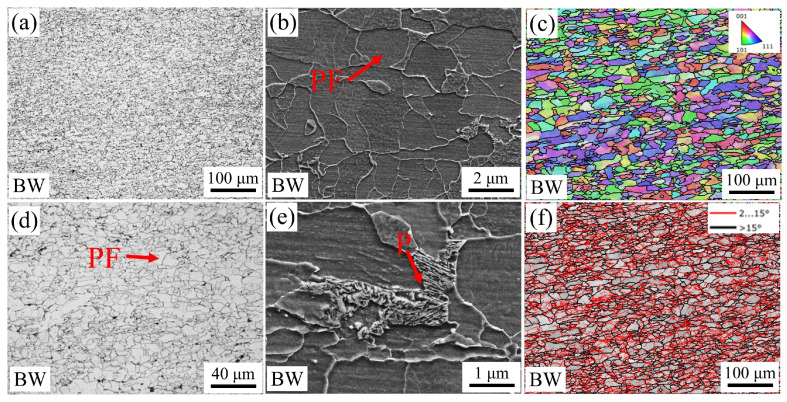
OM, SEM, and EBSD images of the BM. (**a**,**d**) OM, (**b**,**e**) SEM, (**c**) inverseflame pole figure (IPF), (**f**) grain boundary image.

Figure 3 shows the optical microscope (OM), scanning electron microscope (SEM) and electron backscattered diffraction (EBSD) images of X60 steel. As can be seen from Figure 3, the structure of BM is mainly polygonal ferrite (PF), but there is a small amount of lamellar pearlite (P) at the PF grain boundaries (Figure 3e). Grains show elongation in the rolling direction (Figure 3c,f). Grain boundaries with an orientation difference of 2 to 15° are classified as small low-angle grain boundaries (LAGBs), marked by red lines, while those with an orientation difference greater than 15° are classified as high-angle grain boundaries (HAGBs), marked by thick black lines. The proportion of LAGBs in BM is 58.2% and that of HAGBs is 41.8% (Figure 3f).

**Figure 4:**
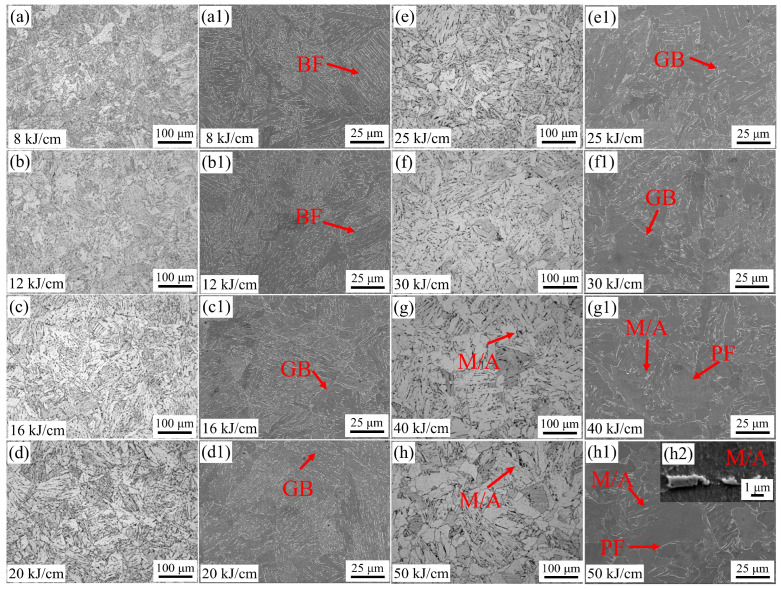
OM and SEM of experimental steel with different heat inputs. (**a**–**h**) OM, (**a1**–**h2**) SEM.

Figure 4 shows the OM and SEM images of the steel samples with different heat inputs. From Figure 4, it can be seen that the microstructure of the steel used in the experiment with a heat input lower than 40 kJ/cm is mainly composed of BF and GB. For the steel samples with 8 and 12 kJ/cm heat input, the microstructure is mainly BF with a small portion of GB. For the steel samples with 16 and 20 kJ/cm heat input, the microstructure is mostly BF with an increasing proportion of GB. For the steel samples with 25 and 30 kJ/cm heat input, the microstructure is mostly GB with a small portion of BF and PF. For the steel samples with 40 and 50 kJ/cm heat input, the microstructure is mainly PF with a small portion of GB. In general, BF, GB, and PF often occur together during the continuous cooling process. As the heat input amount increases, the evolution rule of the CGHAZ microstructure of X60 pipeline steel is that the microstructure changes gradually from BF to GB and PF. The size of the M/A component gradually increases with the increase in heat input.

**Figure 5:**
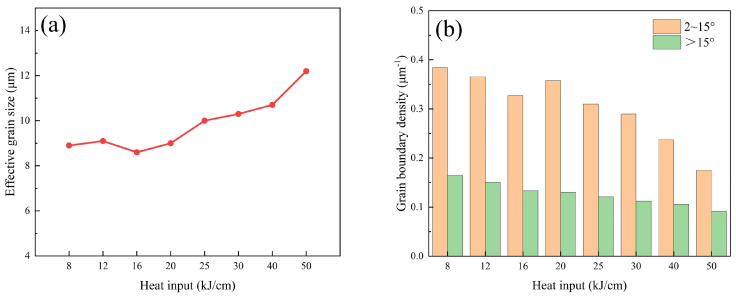
Effective grain size, HAGB and LAGB statistical images. (**a**) effective grain size image, (**b**) image of the proportion of HAGBs and LAGBs.

**Figure 6:**
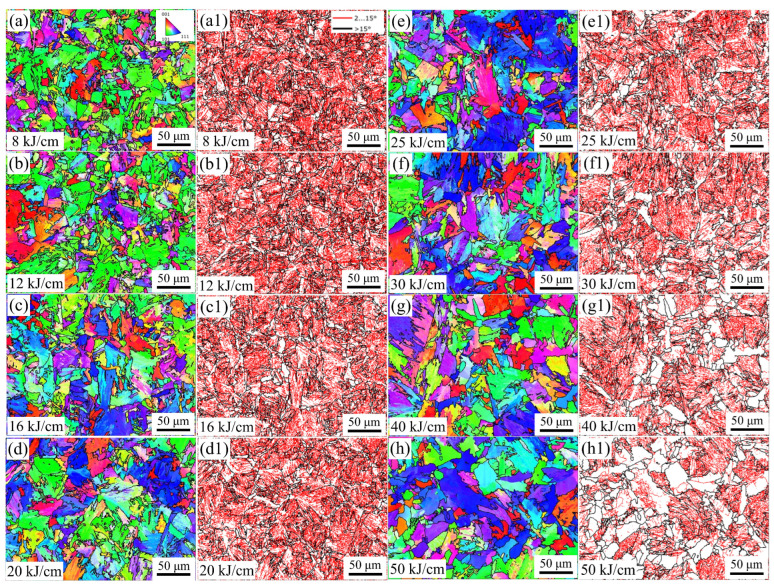
IPF and grain boundary images of experimental steel with different heat inputs. (**a**–**h**) IPF images, (**a1**–**h1**) grain boundary images.

The LAGBs density of the steel sample with 8 kJ/cm heat input is the highest, while that of the steel sample with 50 kJ/cm heat input is the lowest (Figure 5b). The HAGBs density of the steel sample with 8 kJ/cm heat input is the highest. BF has a fine layered structure, arranged in plate-like parallel rows and with LAGB between plates. The GB has a fine block-like structure inside, and the GB is also characterized by the LAGBs. Changes in heat input lead to significant changes in grain size. We used the HKL-Channel 5 (25 December 2025) software to prepare grain size statistics in the central area of the steel samples, with a size of 250 × 250 μm, for different heat input experiments. The grain size of the steel sample with 16 kJ/cm heat input is the smallest and that of the steel sample with 50 kJ/cm heat input is the largest. The minimum grain size is 8.6 μm, and the maximum grain size is 12.2 μm (Figure 5a). The grain size of the steel sample increases overall with the increase in heat input. Figure 6 shows the EBSD images of the steel samples with different heat inputs. The thin red lines represent the LAGBs at 2–15°, and the thin black lines represent the HAGBs greater than 15° (Figure 6a).

### 3.2. Hardness and Low-Temperature Impact Toughness

**Figure 7:**
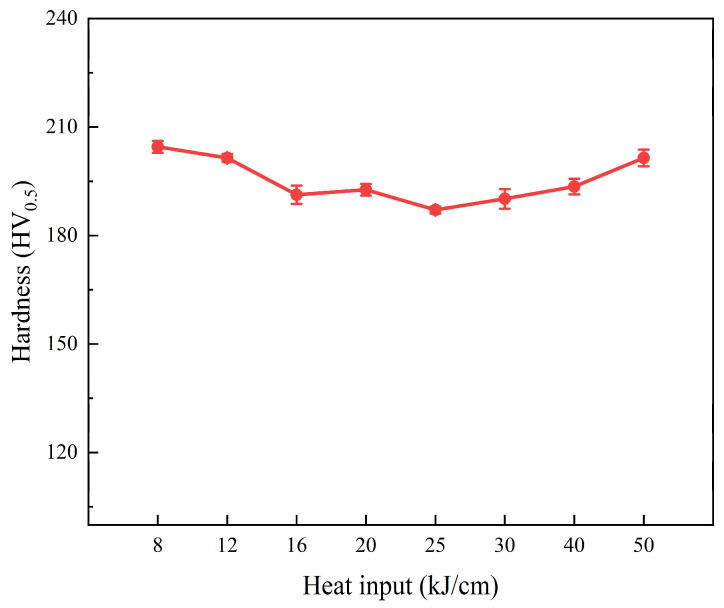
Vickers hardness in experiments with heat input.

Figure 7 shows the hardness of the steel samples under different heat input conditions. The hardness of the steel sample with 8 kJ/cm heat input is the highest, reaching 204.5 HV_0.5_. The hardness of the steel sample with 25 kJ/cm heat input is the lowest, at 187.1 HV_0.5_. The microstructure of the low-heat input samples is mostly BF, which has a plate-like structure and has a high hardness. As the heat input increases, the combination of BF and GB in the microstructure shows a decrease in hardness. At lower heat input levels, as the heat input increases, the proportion of GB in the microstructure also increases. In steel with lower heat input, the M/A components in the GB are finer and more evenly distributed, and they do not significantly enhance the overall hardness of the material. With further increase in the welding heat input, the M/A size in the GB becomes larger, resulting in an increase in the overall hardness of the GB [21,22] because it is not the same, but it can be said that it does not change significantly.

**Figure 8:**
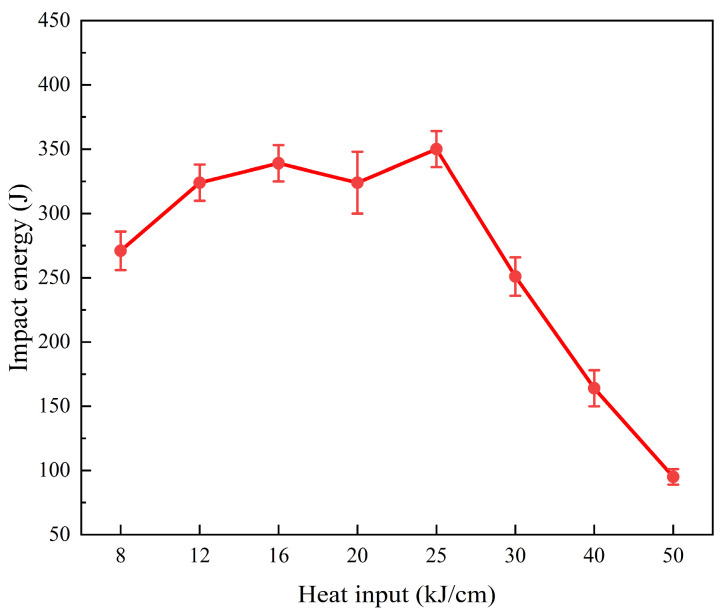
Low-temperature impact toughness (−20 °C) change with the heat input. The error bars in the figure are all standard deviations.

Figure 8 shows the low-temperature impact toughness of the steel samples with different heat inputs. The low-temperature impact toughness of the 25 kJ/cm heat input steel sample at −20 °C is the highest, reaching 350 J. From the literature review, it can be seen that in the pipeline steel, when comparing the same material with a single structure, the low-temperature impact performance is in the following order: GB < BF < PF [23]. The microstructure of the low-heat input steel sample is mainly BF and GB, both of which have good strength and toughness. The microstructure of the higher-heat input steel sample is GB + PF. Although both have good plasticity, the large strength difference between them leads to a decrease in the impact toughness of the steel sample. The increase in grain size with the increase in heat input is the main reason for the significant reduction in the low-temperature toughness.

### 3.3. Hydrogen Damage

#### 3.3.1. HE Sensitivity

**Figure 9:**
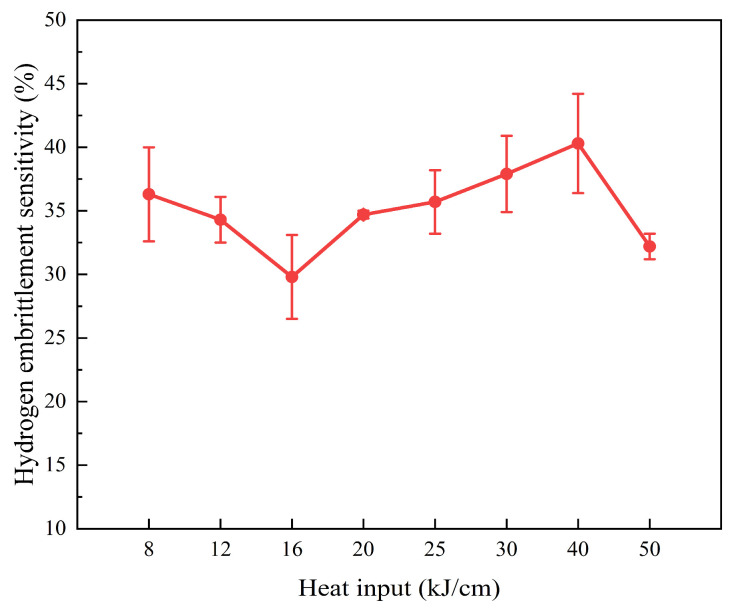
Influence of heat input on HE sensitivity. The error bars in the figure are all standard deviations.

Figure 9 shows the HE sensitivity of the steel samples with different heat input. The HE sensitivity of the steel sample with 16 kJ/cm heat input is the lowest at 29.8%. The HE sensitivity of the steel sample with 40 kJ/cm heat input is the highest at 40.2%. Among them, the increase in heat input, leading to changes in the microstructure type and grain size, is the main reason affecting the HE sensitivity of the experimental steel. BF has a certain inhibitory effect on the crack propagation perpendicular to its platelet bundles, which can reduce the generation of hydrogen-induced cracks [7]. In the lower-heat input experimental steel GB, the M/A component diameter is relatively small, which also helps with resisting crack initiation and propagation and has certain anti-HE performance [24]. At the boundary between GB and PF in the steel subjected to higher-heat input, the stress difference will increase. This is likely to become the location where cracks initiate and propagate, thereby significantly increasing the HE sensitivity of the material. With the increase in heat input, the grain size of the experimental steel increases, and the HE sensitivity also increases. The smaller grain size is the main reason for the low HE sensitivity of the steel sample with 16 kJ/cm heat input.

#### 3.3.2. SSRT Fracture Morphology

**Figure 10:**
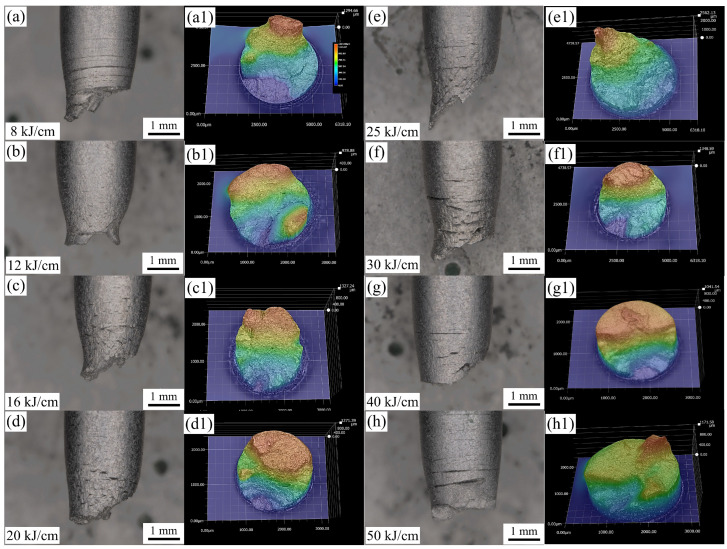
Ultra-depth-of-field extended 3D images. (**a**–**h**) Physical fracture images, (**a1**–**h1**) 3D extended simulation images.

**Figure 11:**
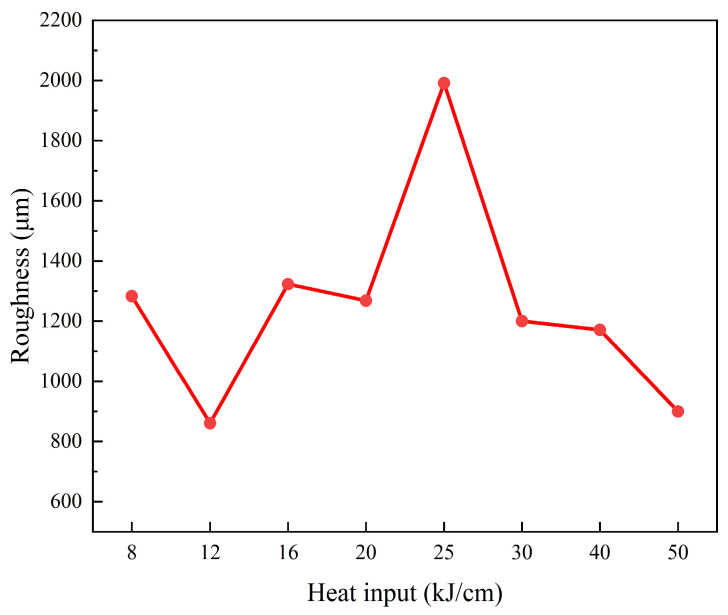
The surface roughness of the fracture of experimental steel SSRT with different heat inputs.

In order to better study the hydrogen damage of the experimental steel, the fracture surface of the experimental steel SSRT was analyzed. Figure 10 shows the ultra-depth extended 3D images of different heat input experimental steels. Whether under higher or lower heat input, circumferential cracks can be observed in the fracture surface of the experimental steel SSRT. The roughness of the SSRT fracture surface of the 25 kJ/cm heat input experimental steel was the highest, reaching 1991.43 μm (Figure 11). The roughness of the SSRT fracture surface of the experimental steel can, to some extent, reflect the HE sensitivity of the material. Generally speaking, the higher the roughness, the higher the HE sensitivity of the material [25]. The 8 kJ/cm and 50 kJ/cm heat input experimental steels had a large number of circumferential cracks and a larger crack depth and width, which was the macroscopic manifestation of their high HE sensitivity. The steel used in the 25 kJ/cm heat input experiment has the characteristics of low hardness and excellent low-temperature impact performance. According to previous experience [26], it should have a lower HE sensitivity, but its HE sensitivity was higher. This issue will be further discussed and studied in the next section.

**Figure 12:**
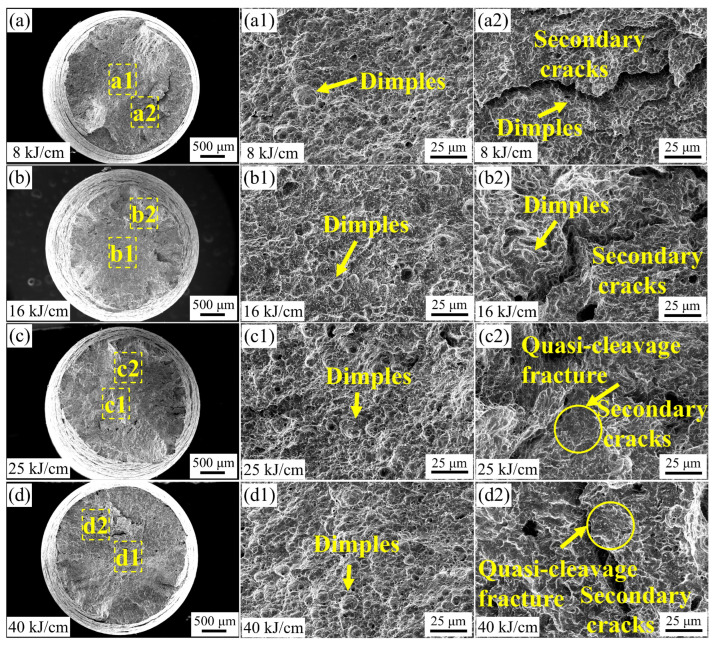
Fracture images of SSRT on experimental steel with different heat inputs after hydrogen filling. (**a**–**d**) Overall fracture morphology, (**a1**–**d1**) the morphology of the central area of the fracture surface, (**a2**–**d2**) secondary cracks at the fracture surface.

Figure 12 shows the representative SEM images of the SSRT test sections of the steel samples with different heat inputs under hydrogen environment. As can be seen from Figure 12(a1–d1), the center positions of the experimental steel under different heat inputs all exhibited ductile fracture. The number and depth of the ductile dimples at the center positions of the SSRT fracture surfaces of the steel samples with 25 kJ/cm and 40 kJ/cm heat inputs are lower than those of the steel samples with 16 kJ/cm heat input. As shown in Figure 12(a2–d2), the secondary cracks in the experimental steel welded with low heat input are accompanied by fine, granular pits. When the heat input increases, obvious quasi-cleavage planes of cleavage fractures appear at the secondary crack locations of the SSRT fracture surfaces of the steel samples. With the increase in heat input, the fracture mode of the steel samples changes, gradually changing from ductile fracture of the steel samples with lower heat input to brittle fracture of the steel samples with higher heat input. The fracture morphology of the SSRT specimens shows that the low-heat input experimental steel mostly undergoes ductile fracture, while in the secondary crack area of the high-heat input experimental steel, quasi-cleavage planes fracture occurs. This result confirms the superior HE resistance of the low-heat input experimental steel and the poor HE resistance of the high-heat input experimental steel (Figure 9).

#### 3.3.3. Hydrogen Micro-Print Techniques

**Figure 13:**
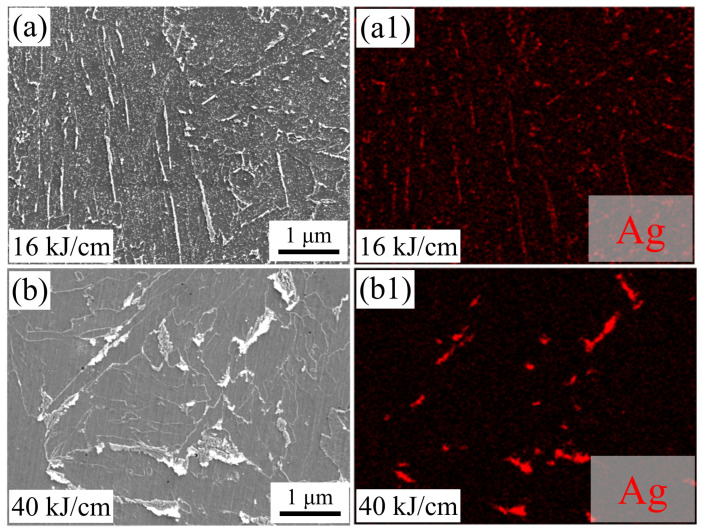
HMT images of test steels with different heat inputs. (**a**,**b**) SEM, (**a1**,**b1**) EDS.

To study the hydrogen damage mechanism of different heat input experiments on steel and observe the distribution of reversible hydrogen in the experimental steel, this paper uses HMTs for characterization. Figure 13 shows the HMT image of the representative experimental steel. The white particles represent silver, which indicate the positions of the reversible hydrogen distribution on the surface of the experimental steel. The spectrogram confirms that the white particles are silver (Figure 13(a1,b1)). The silver particles in the experimental steel are mainly concentrated in the larger M/A components, grain boundaries, and plate structures, indicating that the reversible hydrogen is evenly distributed, with only a small amount of hydrogen located at the grain boundaries and the BF plate interface (Figure 13(a,a1)). For the 40 kJ/cm heat input experimental steel, silver particles mainly aggregate at the boundaries of the carbide cementite-containing transformed M/A components (Figure 13(b,b1)).

## 4. Discussion

Different heat inputs mainly affect the material’s properties by altering the microstructure. Hydrogen atoms diffuse to the highly sensitive positions within the material through specific pathways (grain boundaries, dislocations, second phases, and inclusions), which leads to crack initiation and propagation, thereby triggering the HE mechanism to accelerate the material’s failure [27,28].

**Figure 14:**
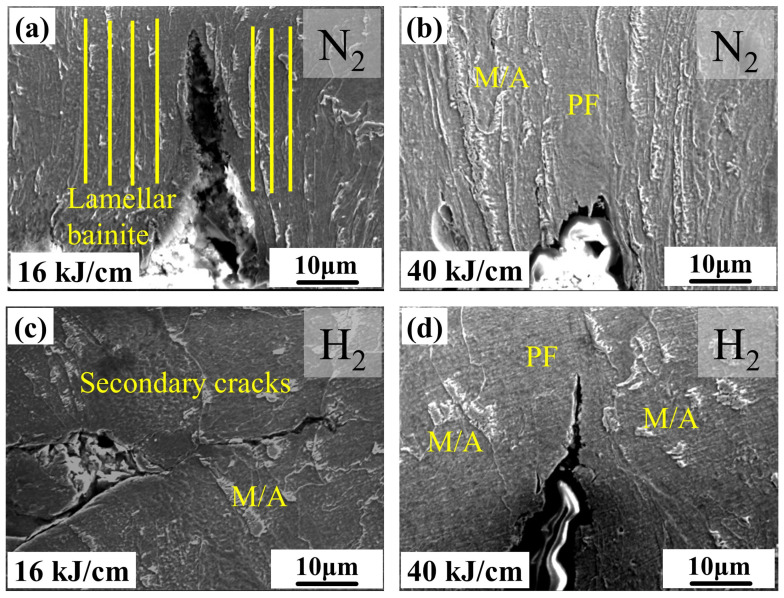
Crack propagation images of tensile fracture surface of experimental steel with different heat inputs. (**a**,**b**) Crack propagation in a nitrogen environment, (**c**,**d**) crack propagation in a hydrogen environment.

During the cooling process after welding, the changes in the microstructure of the pipeline steel within the time range of t_8/3_ (the time required for the steel to cool from 800 °C to 300 °C) are the key factor influencing the performance of the steel. When the heat input is low, the rich carbon austenite cools and transforms into plate BF. In a nitrogen environment with 16 kJ/cm and 40 kJ/cm heat input, the main cracks of the experimental steel are parallel to its strip structure, indicating that BF effectively hinders the crack propagation perpendicular to its strip structure [7]. Combined with Figure 13, the secondary cracks of the experimental steel SSRT fracture occur at the degraded M/A component (Figure 14a), with a small presence of hydrogen at the interface of the strip bundle (Figure 13), suggesting that when the experimental steel microstructure is dominated by BF under low heat input, the hydrogen distribution is relatively uniform, and the material’s anti-HE performance is high. When the heat input increases, the diffusion of carbon has sufficient driving force. The BF continuously grows to enclose the rich carbon austenite, and the remaining island-shaped rich carbon austenite is surrounded by bainite [29,30]. In the SSRT experiment of the experimental steel with 40 kJ/cm heat input, due to the hardness difference between the combination of GB + PF and BF + GB being greater than that of PF + GB and the low hindrance effect of the coarse-grained PF on crack propagation [31], the cracks are prone to pass through the PF grains and undergo transcrystalline fracture, resulting in a sharp decline in the material’s anti-HE performance. During the tensile process, non-coordinated deformation of the different phases forms stress concentration and local plastic deformation at the interface. At the secondary crack location of the SSRT fracture of the experimental steel with 40 kJ/cm heat input, a quasi-cleavage fracture characteristic appears (Figure 12(d2)), and this difference is also the main reason for the sudden drop in its low-temperature impact toughness. Compared to PF, both BF and GB have a higher HAGB density, have a certain hydrogen-capturing ability, and can effectively prevent crack propagation. In the experimental steel with a high heat input, the radius of the M/A component is larger, and there is a significant stress difference between the M/A component and the matrix. This aggravates the process of stress concentration and plastic deformation. Hydrogen is prone to accumulate at the M/A component, triggering the HELP mechanism, and the material fails.

**Figure 15:**
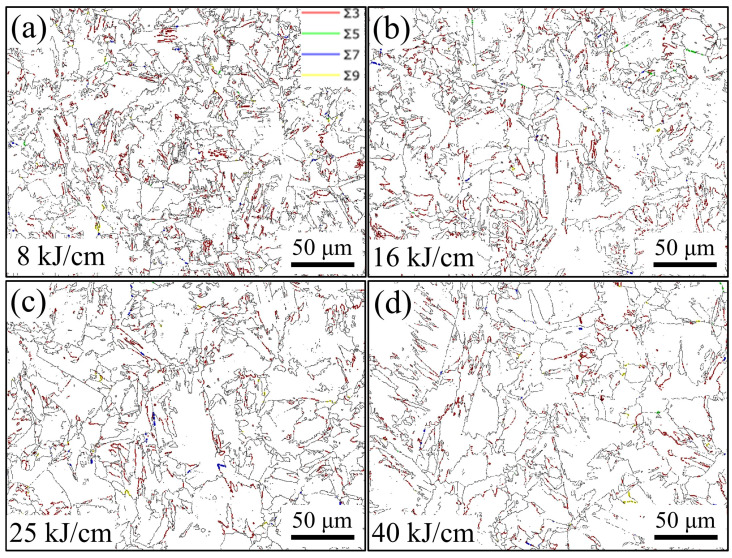
Grain boundaries distribution. Steel subjected to different heat input experiments (**a**) 8 kJ/cm; (**b**) 16 kJ/cm; (**c**) 25 kJ/cm; (**d**) 40 kJ/cm.

**Figure 16:**
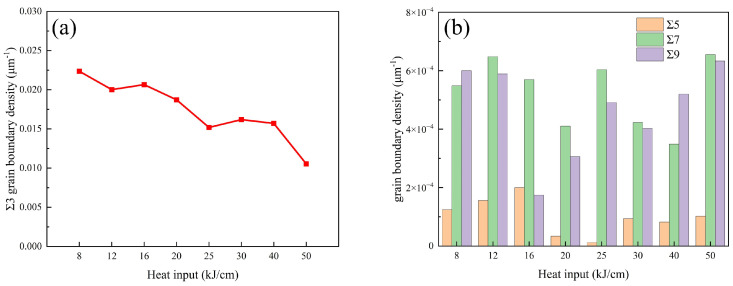
(**a**) Σ3 grain boundary density, (**b**) histogram of Σ5, Σ7, and Σ9 grain boundary densities.

Grain boundaries are generally regarded as low-energy diffusion paths for hydrogen, which can enhance hydrogen diffusion and reduce hydrogen concentration within the region [29,32]. Some special grain boundaries have a crucial impact on the HE resistance of the experimental steel. As shown in Figure 15, the density of Σ3 grain boundaries is significantly higher than that of other special grain boundaries. The density of Σ3 grain boundaries in the experimental steel with 25 kJ/cm and 40 kJ/cm heat input is significantly lower than that in the experimental steel with 16 kJ/cm heat input. Σ3 grain boundaries can effectively accelerate hydrogen diffusion when the local hydrogen concentration at the grain boundaries of the experimental steel is high, adjust the hydrogen distribution of the experimental steel, and prevent the occurrence of local hydrogen enrichment in the experimental steel. Σ3 grain boundaries not only hinder hydrogen-promoting dislocation migration and reduce the risk of triggering the HE mechanism, but also effectively prevent crack propagation along the grain boundaries in body-centered cubic experimental steel. The difference in Σ3 grain boundary density leads to significantly higher HE sensitivity of the experimental steel with 25 kJ/cm and 40 kJ/cm heat input than that of the experimental steel with 16 kJ/cm heat input. The low SSRT roughness of the 25 kJ/cm heat input experimental steel and the high SSRT roughness of the 40 kJ/cm heat input experimental steel also confirm this view (Figure 10(e,e1)). Zhang et al. [33] found that on the same grain boundaries, Σ3 grain boundaries have uniqueness and are less prone to fracture compared to other grain boundaries. Σ3 grain boundaries are likely to act as a rapid hydrogen diffusion channel, thereby reducing the hydrogen concentration in the grain boundary region and ultimately improving the material’s HE resistance. On the one hand, some studies have also shown that Σ3 grain boundaries can reduce the HE resistance of body-centered cubic experimental steel [34,35]. On the other hand, there is no clear conclusion regarding the influence of Σ3 grain boundaries on the HE sensitivity of body-centered cubic experimental steel. The experimental results of this study are more in line with the view that Σ3 grain boundaries can improve the HE resistance of body-centered cubic experimental steel (Figure 16a).

Different types of grain boundaries have varying effects on the HE sensitivity of the material. Among them, the Σ9 grain boundaries are mostly located near the Σ3 grain boundaries. The diffusion speed of hydrogen in the Σ3 grain boundaries is faster than that in the Σ9 grain boundaries, which easily leads to local enrichment of hydrogen in the Σ9 grain boundaries. The steel with 16 kJ/cm heat input experiment has a low density of Σ9 grain boundaries and good anti-HE performance (Figure 16b). Even though there are more dislocation structures in Σ5, Σ7, and Σ9 grain boundaries, this dislocation structure is a typical feature of plastic deformation and helps the alloy release energy and maintain structural stability under stress [33]. However, the density of these special grain boundaries is extremely low, and their protective effect is very limited.

In conclusion, the microstructure type and the grain size are the most critical factors affecting the HE resistance of the experimental steel. The Σ3 grain boundaries can hinder crack propagation, accelerate hydrogen diffusion rate, and reduce the risk of local hydrogen enrichment. Under the condition of meeting the welding requirements, with a heat input of 16 kJ/cm used for welding, the X60 pipeline steel CGHAZ has fine grain size and high Σ3 grain boundary density, thus exhibiting excellent HE resistance. In order to provide data support and theoretical guidance for the selection and optimization of welding processes for future large-diameter, high-pressure hydrogen transmission X60 pipeline steel was explored.

## 5. Conclusions

This paper investigates the influence of welding heat inputs on the hydrogen embrittlement (HE) sensitivity of the coarse-grained heat-affected zone (CGHAZ) in X60 pipeline steel. From the perspectives of microstructure and mechanical properties, the correlation between heat input and HE sensitivity was analyzed. The following conclusions were drawn:

(1) As the welding heat input increases, the microstructure evolution of CGHAZ follows the pattern of transformation from bainitic ferrite (BF) to granular bainite (GB) and finally to polygonal ferrite (PF), with the grain size continuously increasing. The combination of BF and GB has the best anti-HE performance, and the HE sensitivity is 29.8%.
(2) When the heat input is low, the reversible hydrogen mainly accumulates at the grain boundaries and the interface between the BF plates, while the content of irreversible hydrogen is relatively low. However, when the heat input is high, the reversible hydrogen mainly gathers around the M/A components.
(3) The steel with 16 kJ/cm heat input exhibited the best resistance to HE sensitivity, which was attributed to the composite structure of BF + GB, the relatively fine grain size, and the high density of Σ3 grain boundaries. The Σ3 grain boundaries can accelerate the diffusion of hydrogen atoms and reduce the risk of local hydrogen enrichment.

## Figures and Tables

**Table 1:** Chemical compositions in wt.% of steel BM.

Number	C	Si	Mn	Cu	Cr	Ni	Ti	*p*	S	Fe
BM	0.038	0.260	0.81	0.15	0.21	0.16	0.017	≤0.002	≤0.001	Bal.

**Table 2:** The basic mechanical properties of steel BM.

Number	Tensile Strength (Rm), MPa	Yield Strength (Rp_0.2_), MPa	Elongation After Fracture (A), %	Reduction in Area (Z), %
BM	530 ± 5	459 ± 7	29.0 ± 1	78 ± 1

## Data Availability

The original contributions presented in this study are included in the article. Further inquiries can be directed to the corresponding authors.
